# Indoxyl sulfate in uremia: an old idea with updated concepts

**DOI:** 10.1172/JCI155860

**Published:** 2022-01-04

**Authors:** Anders H. Berg, Sanjeev Kumar, S. Ananth Karumanchi

**Affiliations:** 1 Department of Pathology and; 2 Medicine and Biomedical Sciences, Cedars-Sinai Medical Center, Los Angeles, California, USA.; 3 Deparment of Medicine, Beth Israel Deaconess Medical Center and Harvard Medical School, Boston, Massachusetts, USA.

## Abstract

Patients with end-stage kidney disease (ESKD) have increased vascular disease. While protein-bound molecules that escape hemodialysis may contribute to uremic toxicity, specific contributing toxins remain ambiguous. In this issue of the *JCI*, Arinze et al. explore the role of tryptophan metabolites in chronic kidney disease–associated (CKD-associated) peripheral arterial disease. The authors used mouse and zebrafish models to show that circulating indoxyl sulfate (IS) blocked endothelial Wnt signaling, which impaired angiogenesis. Plasma levels of IS and other tryptophan metabolites correlated with adverse peripheral vascular disease events in humans. These findings suggest that lowering IS may benefit individuals with CKD and ESKD.

## Impaired angiogenesis and excess vascular complications in uremia

**Figure 1:**
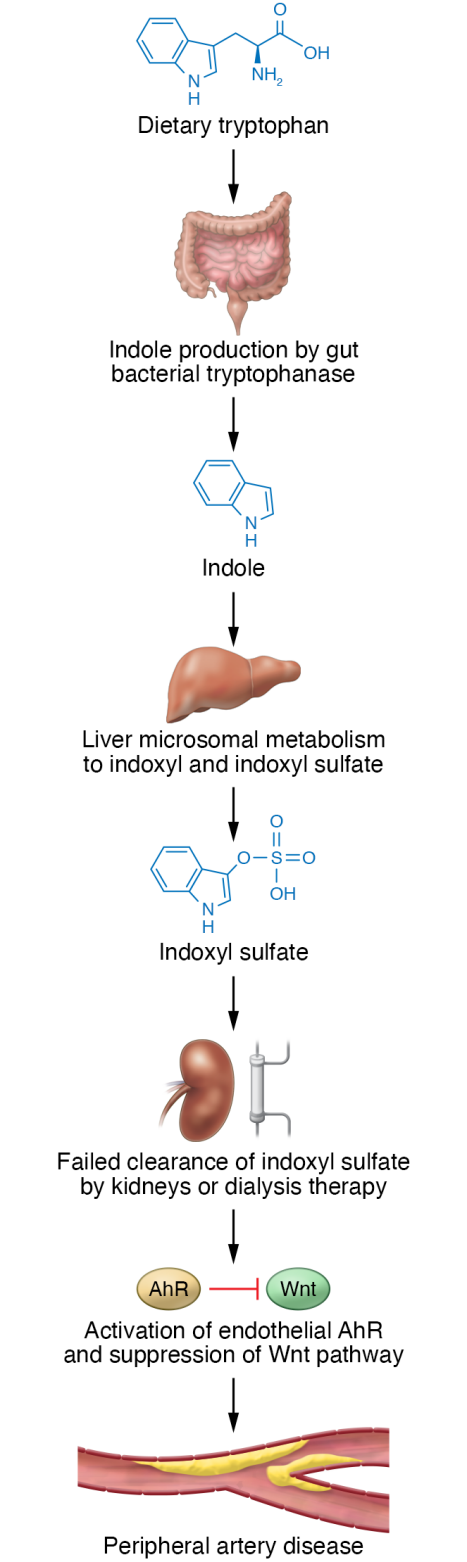
Model of IS synthesis and accumulation in CKD. Gut-residing bacteria produce IS from dietary tryptophan. The hydrophobicity of IS and other tryptophan derivatives favors binding to proteins in the circulation. With CKD, large molecules remain unfiltered by the kidney or dialysis. Consequently, circulating IS accumulates. Increased IS levels activate endothelial AhRs, which suppress the Wnt pathway and damage the vasculature.

The use of chronic dialysis in patients with end-stage kidney disease (ESKD) is one of the miracles of modern medicine with which we can keep patients with kidney failure alive; however, dialysis does not completely remove the many uremic toxins (1). Patients with ESKD have a high burden of cardiovascular morbidity and mortality that is not fully explained by traditional cardiovascular risk factors (2). Traditional hemodialysis technology removes primarily small molecules, but has poor clearance for larger molecules that are often protein bound (3). Therefore, it has been hypothesized that accumulation of protein-bound molecules may contribute to uremia-related damage to the heart, vasculature, and other end organs that are affected in ESKD. To identify pathogenic uremic toxins, many groups have focused on identifying metabolites using sensitive mass spectrometers, and to date, more than 250 such metabolites have been found to accumulate in chronic kidney disease (CKD) and ESKD (4). There is a growing body of evidence that metabolites of tryptophan accumulate in the blood of patients with CKD and ESKD (5). Of these compounds, the most extensively studied molecule is indoxyl sulfate (IS), which is produced from tryptophan by gut bacteria (Figure 1) in a process similar to that of p-cresol sulfate derived from phenyl alanine and tyrosine (6). Previous in vitro studies have suggested that IS is toxic to endothelial cells and impairs angiogenesis by activating the aryl hydrocarbon receptor (AhR) (7, 8).

## A role for tryptophan metabolites in CKD

In this issue of the *JCI*, Arinze et al. provide in vivo evidence in mouse and zebrafish animal models that circulating IS (at concentrations found in humans with CKD) blocks Wnt signaling in the endothelium and impairs angiogenesis in a Wnt ligand–independent fashion (9). Furthermore, they present evidence that plasma levels of IS and other tryptophan metabolites correlate with adverse peripheral vascular disease events in humans.

After ingestion of tryptophan, in addition to its partial conversion to IS in the gut and its absorption and use in protein synthesis, a fraction is metabolized by indole 2,3-dioxygenase to kynurenine, kynurenic acid, and xanthurenic acid, metabolites that are thought to play a modulatory role in central nervous system function. The indole ring structure of IS and the other tryptophan derivatives makes them hydrophobic and largely protein bound in the circulation (10); as a result, they remain unfiltered by the glomerular basement membrane and accumulate in patients with CKD (Figure 1). Their protein-bound status also means that tryptophan derivatives are not readily removed by diffusive hemodialysis procedures. As a result of reduced urinary excretion, IS is markedly increased in CKD and ESKD compared with that in patients with normal kidney function; for example, mean concentrations of IS in patients with CKD and ESKD can be 40 to 90 times higher than those of healthy individuals, making this family of compounds strong candidates as contributors to uremic toxicity (4).

Arinze et al. now provide evidence for a role for tryptophan metabolites in CKD-associated peripheral arterial disease (PAD) (9). The authors showed that tryptophan metabolites that are also known AhR agonists, including IS, kynurenine, kynurenic acid, and xanthurnic acid, suppressed endothelial Wnt signaling by increasing β-catenin polyubiquitination and proteosomal degradation. Notably, proteosomal degradation of β-catenin depends on AhR activation, but not on traditional Wnt receptors, such as the frizzled family of proteins (9). IS inhibited angiogenesis in embryonic zebrafish and reduced neovascularization, capillary density, β-catenin, and vascular endothelial growth factor expression in the hind limbs of mice after ischemic injury. Similarly, mice with adenine-induced CKD treated with hind-limb ischemia that developed seven-fold increases in circulating IS showed decreased endothelial β-catenin expression, decreased neovascularization, and reduced capillary perfusion. Perhaps most intriguingly, treatment of CKD mouse models with an AhR inhibitor effectively rescued the effects of CKD on neovascularization and increased capillary β-catenin and vascular endothelial growth factor expression. These results further indicate the importance of AhR on mediation of the effects of IS and other tryptophan metabolites. Finally, in a small-nested case-control study, the authors found that concentrations of IS and other tryptophan metabolites were higher in CKD patients with PAD who developed adverse limb events compared with those in individuals that had PAD without adverse events. Together, these findings provide compelling evidence that the effects of elevated tryptophan metabolites on AhR and Wnt pathway signals contribute to vascular pathology associated with CKD.

## Conclusions and clinical implications

While the in vivo findings compellingly link IS with PAD, several questions remain unanswered. The hind-limb ischemia model is a comparatively acute model focusing on capillary bed density that may only reflect part of the pathophysiology of PAD associated with CKD. Although increased concentrations of IS have been associated with PAD in hemodialysis patients, IS does not seem to have any substantial association with other cardiovascular outcomes (11). Consequently, other toxins may play notable roles in uremic cardiomyopathy and the cardiovascular disease of CKD. There is growing evidence that protein modifications by urea (protein carbamylation) may play roles in the progression of kidney disease, CKD-associated cardiovascular disease, and mortality (12, 13). Moreover, the pathophysiologic contributions of many other alleged uremic toxins, such as fibroblast growth factor 23 (14) and asymmetric dimethyl arginine (15), deserve further evaluation. Although kynurenine, kynurenic acid, and xanthurenic acid derived from indolamine dioxygenase (IDO) accumulate in CKD and may also activate AhR, studies indicate that kynurenine pathway metabolites have beneficial vascular effects via the orphan G protein–coupled receptor 35 (16). In renal tissue, kynurenine pathway metabolites may promote nicotinamide adenine dinucleotide–positive (NAD) synthesis with related benefit (17). Additional studies are needed to better understand which AhR pathway metabolites promote favorable outcomes and which end products have harmful effects.

Taken together with other published studies, Arinze’s study shows that activation of AhR by IS and other tryptophan metabolites is now implicated in multiple pathophysiologic processes associated with CKD, including glomerular injury, renal fibrosis, atherogenesis and cardiovascular disease, protein energy wasting, renal anemia, loss of bone mineralization, and cognitive dysfunction (5). Findings by Arinze et al. raise exciting possibilities of studying the IS pathway as a therapeutic target for patients with CKD and ESKD. Selective targeting of the kynurenine biosynthetic pathway to reduce toxic end products without interfering with NAD biosynthesis or reduction of intestinal conversion of tryptophan into IS may provide therapeutic avenues for people with kidney disease. A recent report demonstrated that a sulfur amino acid–enriched diet can reduce indole and IS production in a CKD animal model, which in turn was associated with reduced CKD progression (18). Other possibilities include lengthening the dialysis sessions using high-flux membranes or using adsorption treatments (additive charcoal to dialysate) to help efficiently remove protein-bound molecules, such as IS (19, 20). During the last five years, there has been a tremendous interest in the renal community in innovating dialysis treatments. The American Society of Nephrology working with the Food and Drug Administration has launched the Kidney Health Initiative to support innovators in academics and industry to develop improvements for dialysis efficiency and delivery (1). The findings from Arinze et al. justify further studies investigating medical benefits of indole-lowering therapies and monitoring of IS levels in addition to traditional renal biomarkers. We need clinical trials to evaluate whether lowering IS levels and other tryptophan-derived metabolites in CKD and ESKD will lead to improved clinical outcomes.

## Version 1 01/04/2022

Electronic publication
